# Isolation and Identification of Gas‐Producing Spoilage Bacteria in Wasabi Sauce and Effective Methods for Controlling Their Growth

**DOI:** 10.1002/fsn3.71750

**Published:** 2026-09-23

**Authors:** Xiao Liu, Hong Li, Qin‐Guo Xu, Weiwei Wu, Qing‐Dian Han, Yun‐Guo Liu

**Affiliations:** ^1^ Linyi University Linyi China; ^2^ China National Accreditation Service for Conformity Assessment Beijing China; ^3^ Shandong Executive Chef Food Co., Ltd Linyi China

**Keywords:** gas‐producing spoilage microbe, isolation and identification, minimum inhibitory concentration, wasabi sauce

## Abstract

The presence of gas‐producing spoilage bacteria in wasabi sauce can lead to aerogenesis defects, severely compromise product quality, and cause potential food safety hazards—issues that have become increasingly urgent within the condiment industry. The purpose of this study is to analyze the quality changes of wasabi sauce after gas production within its shelf life, isolate and identify the gas‐producing bacteria, and propose effective methods to inhibit their growth. The gas‐producing spoilage microbe was isolated in anaerobic conditions and identified as *Bacillus licheniformis* QJL3. This strain was tolerant to high salt concentrations and moderate heat, but sensitive to highly acidic conditions: it could survive after being treated at 4°C and 55°C for 10 min, grew at pH as low as 3.8, and its growth was completely suppressed at 14% salt concentration. Inhibition analysis indicated that the growth of *B. licheniformis* QJL3 in Yeast Extract Peptone Dextrose Adenine Medium liquid medium could be effectively inhibited when the salt concentration reached 8%, and the minimum inhibitory concentration of potassium sorbate was 0.2 mg/mL. These findings offer valuable insights for wasabi sauce manufacturers to implement targeted microbial control measures, thereby improving product stability and ensuring food safety.

## Graphical abstract

Food package bulging, a prevalent spoilage issue in dairy products, beverages, and condiments, is predominantly attributed to gas‐producing spoilage bacteria. These contaminants originate from multiple pathways: raw material contamination, processing cross‐contamination, packaging material leaching, airborne microbial ingress, and inadequate control of storage temperature and humidity. Advanced microbiological and molecular biological techniques enable precise identification of key gas‐producing species. Targeted control strategies, including optimized thermal processing and antimicrobial interventions, are subsequently implemented to suppress microbial proliferation and gas generation, thereby mitigating package bulging, extending product shelf life, and safeguarding food safety.

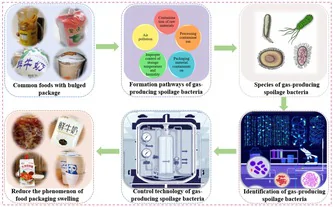

**Abbreviations**
ABTS: 2,2′‐Azinobis‐(3‐ethylbenzthiazoline‐6‐sulphonate)
CFU: colony‐forming units
DPPH: 2,2‐Diphenyl‐1‐picrylhydrazyl
MIC: minimum inhibitory concentration
PCA: plate counting agar medium
PCA: principal component analysis
RBM: Rose Bengal medium
TSA: tryptone soy agar
YPDA: yeast extract peptone dextrose adenine medium

## 1 Introduction

Wasabi sauce, also known as green mustard or horseradish, is a condiment sauce primarily made from the root of the horseradish plant ( *Armoracia rusticana* ) (Morimitsu et al. 2000). It naturally contains glucosinolates, which, upon exposure to water under appropriate temperature and pH conditions and in the presence of the enzyme myrosinase, are hydrolyzed into isothiocyanates. Isothiocyanates are the source of the unique aroma and pungent spiciness of wasabi sauce, which can stimulate the secretion of saliva and gastric juice, thereby enhancing appetite. Additionally, wasabi sauce is known for its strong detoxifying properties, including the ability to neutralize toxins from fish and shellfish. As a result, it is traditionally served with sashimi and sushi in Japanese cuisine, not only to impart flavor but also to deodorize and provide antimicrobial benefits.

Wasabi sauce typically exhibits a relatively low pH of 4.8–5.2 and a high salt concentration of approximately 10%–12% (w/v), conditions that are generally unfavorable for bacterial growth. Additionally, the production process of wasabi sauce is extremely simple and typically does not involve a prolonged microbial fermentation process, thereby minimizing the risk of cross‐contamination commonly associated with fermented products. Moreover, the horseradish extract—a key ingredient in wasabi sauce—can kill or inhibit food spoilage bacteria (Kim et al. 2015). In recent years, growing public health awareness and the implementation of dietary policies have driven a global trend toward reduced salt consumption (Jelakovic et al. 2024). The Chinese government has set a target of reducing per capita salt intake by 20% by 2030 as outlined in the National Nutrition Plan (2017–2030). In response, wasabi sauce producers have begun lowering the salt concentration in their formulations. However, this reduction in salinity has led to sporadic outbreaks of spoilage, most notably characterized by gas production. Such defects can result in product quality issues, including package swelling and sour off‐flavors during storage, ultimately diminishing consumer confidence and causing economic losses (Zhao et al. 2020; Snyder et al. 2016). Hu et al. (2023) found that in soy sauce, salt reduction leads to an increase in the total number of bacteria (by 1–2 orders of magnitude) as well as the population of spoilage bacteria with gas‐producing properties. Therefore, the systematic isolation and identification of gas‐producing bacteria, along with the investigation of their characteristics and control measures, are critical for enhancing the shelf‐life stability and food safety of wasabi sauce.

Aerogenesis defects are a prevalent issue in the food industry and have been reported in a variety of products, including chili sauce (Zheng et al. 2020), bean paste (Niu et al. 2018), sufu (Qu et al. 2022), soy sauce (Liu et al. 2013), and cheese (Fusieger et al. 2022). These defects refer to undesirable spoilage phenomena—such as gas production, package swelling, or even rupture—caused by gas‐producing microbial contamination during food production, storage, or fermentation (Sheehan 2011). At present, many spoilage bacteria that cause aerogenesis defects in food have been isolated and identified, and a range of control strategies has been developed accordingly. For example, Zhang et al. isolated three strains of gas‐producing bacteria, *Bacillus amyloliquefaciens* , *Bacillus* sp., and *Bacillus subtilis* , from swollen bagged soy sauce (Zhang et al. 2023). They demonstrated that 0.22 μm inorganic microfiltration, sterilization at 121°C for 2 min, and the addition of 1 g/kg potassium sorbate effectively inhibited these strains and mitigated package bloating. During sufu fermentation, *W. confusa* and *W. koreensis* were identified as the primary gas‐producing bacteria. In addition, some studies have found *Lactobacillus acidus R‐19056*, *Sinus sylvae‐CICC10181*, and *Bacillus licheniformis CCMMB912* to be the primary gas‐producing spoilage bacteria in chili sauce, and their growth was effectively inhibited by the combined use of lactic acid streptococcin and salt (Niu et al. 2020). Microbial gas production negatively impacts food quality and shelf life. To address this issue, various strategies have been employed, such as thermal treatment, addition of bacteriocins and preservatives (potassium sorbate or sodium benzoate) (Kilic‐Akyilmaz et al. 2023; Abid et al. 2021). However, the efficacy of these interventions varies widely, depending on the specific spoilage organisms involved and the environmental conditions. Therefore, it is essential to comprehensively evaluate and validate strategies to avoid gas production problems in wasabi sauce.

In this study, to elucidate the cause of aerogenesis defects in wasabi sauce, potential gas‐producing bacteria were isolated by culturable method and Dulbecne tubule test. These bacteria were then identified by traditional identification methods and molecular biology techniques, so as to address the issue of package swelling in the commercial wasabi sauce. In addition, the environmental tolerance of the identified spoilage bacteria was assessed, and the inhibitory effects of bacteriocins, preservatives, and salt concentrations on their growth were systematically evaluated.

## 2 Materials and Methods

### 2.1 Materials

Both normal wasabi sauce and wasabi sauce with bulged package samples were sourced from a condiment factory in Shandong, China. The three normal wasabi sauce samples were labeled ZC1, ZC2, and ZC3, with production dates of April 8, July 23, and November 16, 2023, respectively; the three gas‐producing samples with bulged packages were labeled CQ1, CQ2, and CQ3, and all were produced on March 2, 2023 (Table 1).

**TABLE 1:** Wasabi sample information.

Sample number	Origin	Date of manufacture	Shelf life	Storage temperature
ZC1	A food company in Shandong	April 8, 2023	18 months	Ambient temperature
ZC2		July 23, 2023		
ZC3		November 16, 2023		
CQ1		March 2, 2023		
CQ2		March 2, 2023		
CQ3		March 2, 2023		

### 2.2 Determination of Physicochemical Indicators and Sensory Parameters

The silver nitrate titration method (Rasmussen et al. 2010) was used to determine the salt concentration in wasabi sauce samples. The total acid content and amino acid nitrogen content in wasabi sauce samples were determined by the sodium hydroxide titration method, expressed in lactic acid equivalent (Hao et al. 2017). Briefly, filtrates from the wasabi sauce samples were titrated to a pH of 8.2 using 0.05 mol/L NaOH to quantify total acidity based on titrant consumption. Subsequently, 10.0 mL of formaldehyde solution was added to the sample and mixed thoroughly. The sample was then further titrated to a pH of 9.2 with the standard NaOH titrant, and the amino nitrogen content of the wasabi sauce was determined based on the volume of the standard titrant used (Zhang et al. 2016). The content of soluble sugar was determined using a plant soluble sugar content detection kit (anthrone colorimetric method) (Solarbio Science & Technology Co. Ltd., Beijing, China) (Zeng et al. 2016; Li et al. 2019). The pH value and moisture content of the wasabi sauce samples were measured using a pH meter and the direct drying method, respectively. The protein content in wasabi sauce samples was determined using the combustion method as described by Wei and Wang (2023). In brief, 0.1 g of the wasabi sauce sample was weighed, wrapped in tin foil, and placed on a sample tray. The sample was fully combusted in the combustion reactor (900°C–1200°C). The combustion products (NO_*x*_) were transported to the reduction furnace (800°C) by the carrier gas (nitrogen). Subsequently, the content of nitrogen was measured after the reduction of nitrogen production.

For sensory evaluation, a clean, well‐lit, and odor‐free room was used. Twelve trained sensory evaluation experts assessed the samples. A 10.0 g sample of wasabi sauce was weighed and placed in a petri dish, then stirred and flattened with a glass rod. The sensory characteristics of the wasabi sauce, including appearance, color, aroma, taste, and the presence of visible impurities, were observed under standardized lighting (Liu, Gong, et al. 2025; Liu, Liu, et al. 2025). Taste assessments included the six basic modalities—sweet, sour, salty, bitter, pungent, and astringent—with normal samples serving as controls.

### 2.3 Determination of Antioxidant Components and Activity

#### 2.3.1 Determination of Antioxidant Components

According to the instructions of the relevant test kit (Beijing Solarbio Technology Co. Ltd., China), three antioxidant components—total phenols, flavonoids, and ascorbic acid in wasabi sauce were determined. The absorbance values were measured at 760, 470, and 525 nm, respectively (Wang et al. 2020).

#### 2.3.2 Determination of 2,2‐Diphenyl‐1‐Picrylhydrazyl (DPPH) Radical Scavenging Activity

The DPPH radical scavenging activity of flavonoids in wasabi sauce was assessed using a slightly modified version of the method described by Li et al. (2021). At room temperature, 40 μL of the wasabi sauce sample solution was mixed with 0.2 mL of DPPH (0.1 mM methanol solution). Thereafter, the mixture was incubated at 25°C in the dark for 30 min, and the absorbance of the mixture solution was recorded at 517 nm and denoted as *A*_*i*_. Meanwhile, the DPPH solution was replaced with a methanol solution, and its absorbance was determined and expressed as *A*_*j*_. In addition, 40 μL of methanol solution was added to 0.2 mL of DPPH solution, and its absorbance was recorded as *A*_0_. The DPPH scavenging ability was calculated as follows:

$$I\%=\left(1-\frac{A_{i}-A_{j}}{A_{0}}\right)\times 100\%$$

where *A*_*i*_ indicates the absorbance of the sample solution; *A*_*j*_ represents the absorbance without DPPH (replacing the DPPH methanol solution with an equal volume of methanol); and *A*_0_ indicates the absorbance without the sample (the sample solution is replaced with an equivalent amount of methanol).

#### 2.3.3 Determination of 2,2′‐Azinobis‐(3‐Ethylbenzthiazoline‐6‐Sulphonate) (ABTS) Radical Scavenging Activity

ABTS radical scavenging activity was measured with reference to Bilal et al. (2016), with slight modifications. A total of 96 mg ABTS was dissolved in 20 mL of deionized water, and 5 mL of 2.45 mM potassium persulfate solution was added. The mixture was kept in the dark at room temperature for 12 h to obtain the ABTS radical solution. The ABTS radical solution was diluted approximately 70 times with methanol, and its absorbance was measured at 734 nm to be 0.70 ± 0.02. Then, 40 μL of the wasabi sauce sample solution was added to 200 μL of the aforementioned ABTS radical solution. After incubation in the dark at 30°C for 6 min, the absorbance of the sample at 734 nm was measured using a microplate reader and denoted as *A*_test_. At the same time, the ABTS solution was replaced with a methanol solution; its absorbance was determined and denoted as *A*_control_. In addition, 40 μL of methanol was added to 200 μL of the ABTS radical solution, and its absorbance was recorded as *A*_blank_. The ABTS scavenging ability was calculated as follows:

$$D\%=\frac{\left[A_{\text{blank}}-\left(A_{\text{test}}-A_{\text{control}}\right)\right]}{A_{\text{blank}}}\times 100\%$$

#### 2.3.4 Determination of Hydroxyl Radical Scavenging Capacity

A 0.1 g portion of the wasabi sauce sample was homogenized with 1 mL of extraction solvent and centrifuged at 4°C for 10 min. The resulting supernatant was collected for analysis. The absorbance at 536 nm was measured according to the determination steps (Yang et al. 2020). The absorbance values of the blank tube, control tube, and test tube were denoted as *A*_blank_, *A*_control_, and *A*_test_, respectively. The formula for calculating the hydroxyl radical scavenging rate is as follows:

$$S\%=\left(A_{\text{test}}-A_{\text{control}}\right)÷\left(A_{\text{blank}}-A_{\text{control}}\right)\times 100\%$$

### 2.4 Isolation of Spoilage Bacteria in Gas‐Producing Wasabi Sauce

A total of 25 g of the swollen wasabi sauce was mixed with 225 mL of sterile physiological saline and stirred until thoroughly homogenized. The stirred wasabi sample solution was then subjected to serial dilution to achieve concentrations ranging from 10^−3^ to 10^−6^. The diluted 100 μL solution was applied to four agar plates: Rose Bengal medium (RBM), plate counting agar medium (PCA), tryptone soy agar (TSA), and yeast extract peptone dextrose adenine medium (YPDA). The RBM medium plates were placed in a 30°C incubator for 5 days for the aerobic cultivation of molds; the rest of the medium was placed in a 37°C incubator for aerobic culture. Whereas anaerobic growth of strains on agar plates is carried out in anaerobic tanks. After 48 h of incubation, the colony‐forming units (CFU) (if CFU < 30/mL, exact counts were noted) and different colony morphologies were observed. Colonies grown on agar plates after aerobic and anaerobic incubation were incubated at 37°C for 48 h in the corresponding liquid medium fitted with Durham tubes. According to the methods previously reported (Endoh et al. 2021; Jonghe et al. 2010), the gas‐producing ability of the bacterial strains was tested using Durham tubes. If the Durham tube was filled with gas after incubation, it indicated that the strain had a strong gas‐producing ability.

### 2.5 Morphological, Physiological, and Biochemical Analysis

The strains with gas‐producing ability were streaked and cultured on the corresponding agar medium. Based on Bergey's Manual of Systematic Bacteriology, the colony morphology of gas‐producing bacteria after Gram staining was observed with a microscope. Biochemical identification was conducted using an automated microbial identification system (VITEK 2, bioMérieux, France), assessing various biochemical reactions of gas‐producing bacteria, including enzyme reactions, acidification test, assimilation test, antibacterial sensitivity, and sugar utilization ability, to support preliminary species identification.

### 2.6 Molecular Identification

The genomic DNA of gas‐producing bacteria was extracted using a bacterial DNA extraction kit (Solarbio‐Science and Technology Co. Ltd., Shanghai, China), and the forward primer 27F (5′‐AGAGTTTGATCMGGCTCAG‐3′) and the reverse primer 1492R (5′‐GGTTACCTTGTTACGACTT‐3′) were used. Polymerase chain reaction (PCR) was used to amplify the 16S rDNA sequence of gas‐producing bacteria. Primer synthesis and PCR product sequencing were completed by Sangon Biotech Co. Ltd. The 16S‐PCR system (total volume of 25 μL) consisted of 12.5 μL of 2× Premix rTaq, 2 μL each of the 27F and 1492R primers, 2 μL of template DNA, and 6.5 μL of ddH_2_O. The reaction process is as follows: 94°C for 3 min, 42°C for 2 min, 72°C for 3 min (94°C for 45 s, 42°C for 40 s, 72°C for 2 min) × 30 cycles, and finally extended to 72°C for 10 min. The PCR products were purified and sequenced using a circulating purification kit. The 16S rDNA sequence was then submitted to the BLAST (https://blast.ncbi.nlm.nih.gov/Blast.cgi) online software for identification. Subsequently, sequences of 15 different strains were selected, and a phylogenetic tree was constructed using the adjacency method of MEGA version 7.0 (Tamura et al. 2021).

### 2.7 Environmental Tolerance of Gas‐Producing Spoilage Bacteria

The gas‐producing bacteria at a concentration of 1 × 10^6^ CFU/mL were inoculated in YPDA liquid medium to investigate their tolerance under different environmental conditions. The growth of the bacteria was measured by OD_600_ every 4 h. The suspension of gas‐producing spoilage bacteria was treated at different temperatures (4°C, 15°C, 25°C, 37°C, 45°C, 55°C, and 65°C) for 10 min. After cooling, the treated suspensions were spread onto YPDA agar plates. The number of colonies was recorded after overnight incubation at 37°C to determine the temperature tolerance of gas‐producing bacteria. The acid‐tolerant ability of gas‐producing bacteria was determined by incubating the gas‐producing bacteria at a concentration of 1 × 10^6^ CFU/mL in YPDA liquid medium at 37°C at different pH values (2.6, 3.6, 4.6, 5.6, 6.6, and 7.6) for 48 h. The salt tolerance of gas‐producing bacteria was assessed by inoculating gas‐producing bacteria at concentrations of 1 × 10^6^ CFU/mL in different salinities (2%, 4%, 6%, 8%, 10%, 12%, 14%, and 16%) YPDA liquid medium and incubating at 37°C for 48 h. Then, the absorbance of the sample at 600 nm was measured using a microplate reader (Bio‐Tek Instruments Inc., USA).

### 2.8 Inhibitory Effects of Nisin, Preservatives, and Salt on Gas‐Producing Spoilage Bacteria

Given the semi‐solid nature of wasabi sauce, direct monitoring of *B. licheniformis* growth within it is challenging. Therefore, the inhibitory effects of nisin, preservatives, and salt on the growth of *B. licheniformis* were investigated in YPDA medium. Commercial nisin (Yota Bio‐Engineering Co. Ltd., China) was prepared as a stock solution with a concentration of 11,500 IU/mg. Potassium sorbate and sodium benzoate (Sinopharm, Beijing, China) were each dissolved in distilled water to prepare stock solutions with a concentration of 16 mg/mL. All solutions were sterilized by filtration through a 0.22 μm filter membrane and stored at 4°C for later use. The minimum concentration of nisin, potassium sorbate, and sodium benzoate in YPDA liquid medium at 37°C to completely inhibit the growth of gas‐producing spoilage bacteria was defined as the minimum inhibitory concentration (MIC). The nisin and preservatives were diluted to different concentrations in YPDA liquid medium to a final concentration ranging from 0.2 to 8 mg/mL. The gas‐producing spoilage bacteria were incubated on a shaker at 37°C and 180 r/min until the OD_600_ value reached about 0.9. Then, 200 μL of bacterial culture was inoculated into 20 mL of YPDA liquid medium containing different concentrations of nisin and preservatives. The medium containing bacteria and nisin or preservatives was incubated in a shaker at 37°C for 48 h, and OD_600_ values were recorded every 4 h. Growth curves under different conditions were plotted to determine the MICs of these three antimicrobial compounds. To evaluate the effects of nisin and preservatives on spoilage microorganisms under high‐salt conditions. The effects of potassium sorbate and sodium benzoate on the inhibition effect of gas‐producing spoilage bacteria were studied under the condition of 8% salt concentration, and the same method was used to determine MICs. Since nisin exhibited no inhibitory effect on *B. licheniformis* QJL3, it was excluded from further testing.

### 2.9 Statistical Analysis

All experiments were conducted in triplicate, and the results are expressed as the mean ± standard deviation of the three replicates. One‐way analysis of variance (ANOVA) in SPSS 23.0 software was used for data analysis, and Duncan's post hoc test was performed to ensure that the difference was statistically significant (*p* < 0.05). Principal component analysis (PCA) and cluster analysis were performed using Origin 2024b (OriginLab, USA) and SIMCA 13 (Umetrics, Sweden), respectively. Phylogenetic trees were constructed using MEGA 11.0 software.

## 3 Results and Discussion

### 3.1 Physicochemical and Sensory Property Comparison

Sensory and physicochemical properties are important indicators for evaluating food quality. The physicochemical and sensory parameters of normal and aerogenic wasabi sauce are shown in Table 2. Significant differences were observed in sensory characteristics between the bulged‐package wasabi sauce and the normal counterpart, though no notable differences were found in terms of visible impurities. Regarding packaging appearance, aerogenic wasabi sauce samples exhibited visible swelling due to internal gas accumulation, whereas normal samples did not. In terms of color, normal wasabi sauce appeared bright green, while bloated samples exhibited a darker, brownish hue. This may be due to enzymatic or non‐enzymatic reactions occurring during processing or storage, leading to the degradation, oxidation, or other chemical changes of pigments, which alter the color appearance of the product (Lertsiri et al. 2010; Sugawara et al. 2007). In terms of odor, the pungent smell of aerogenic wasabi sauce was weakened, and there was a putrid odor but no musty odor, while the normal wasabi sauce had a pungent and spicy aroma. Neither normal wasabi sauce nor wasabi sauce with a bulged package has mold or moldy odors, indicating that wasabi sauce is unlikely to be contaminated by mold in the air. Niu et al. (2020) found that compared with the normal samples, the bulged chili sauce samples were significantly darker, accompanied by an unpleasant odor, and no hyphae or other phenomena associated with mold contamination were observed. Regarding taste, wasabi sauce with a bulged package has a slightly sour and astringent taste and has a weaker pungent taste, while normal wasabi sauce has a strong pungent flavor and regular granules.

**TABLE 2:** Sensory and physicochemical properties of normal (marked ZC1, ZC2, and ZC3) and gas‐formed wasabi (marked CQ1, CQ2, and CQ3).

Property/sample No.		ZC1	ZC2	ZC3	CQ1	CQ2	CQ3
Sensory property	Packing status	Normal	Normal	Normal	Swell	Swell	Swell
	Color	Bright green	Bright green	Bright green	Brown	Brown	Brown
	Smell	Irritating pungent spiciness	Irritating pungent spiciness	Irritating pungent spiciness	The spiciness weakens, spoiled odor	The spiciness weakens, spoiled odor	The spiciness weakens, spoiled odor
	Taste	Cool, pungent	Cool, pungent	Cool, pungent	Astringent	Sour and astringent	Sour and astringent
	Impurity	No	No	No	No	No	No
Physicochemical property	Salt content (g/100 g)	11.493 ± 0.025^a^	11.550 ± 0.020^a^	11.487 ± 0.086^a^	11.257 ± 0.031^b^	11.323 ± 0.031^b^	11.217 ± 0.025^b^
	Total acid (g/kg)	1.350 ± 0.450^b^	1.113 ± 0.226^b^	1.273 ± 0.108^b^	2.250 ± 0.450^a^	1.943 ± 0.266^ab^	2.533 ± 0.247^a^
	Amino acid nitrogen (g/100 g)	0.026 ± 0.004^b^	0.030 ± 0.002^ab^	0.033 ± 0.002^a^	0.014 ± 0.000^c^	0.015 ± 0.002^c^	0.016 ± 0.002^c^
	Soluble sugars (mg/g)	211.126 ± 5.352^a^	201.825 ± 12.787^a^	194.436 ± 7.747^a^	181.936 ± 9.621^a^	191.176 ± 14.183^a^	192.022 ± 11.910^a^
	pH	5.040 ± 0.053^b^	4.957 ± 0.125^b^	5.100 ± 0.044^b^	5.313 ± 0.015^a^	5.337 ± 0.015^a^	5.310 ± 0.010^a^
	Moisture content (g/100 g)	19.809 ± 0.085^a^	19.763 ± 0.056^a^	19.778 ± 0.076^a^	19.296 ± 0.150^b^	19.124 ± 0.020^b^	19.202 ± 0.321^b^
	Protein (g/100 g)	4.2 ± 0.06^a^	3.96 ± 0.08^a^	3.93 ± 0.11^a^	3.32 ± 0.08^b^	2.92 ± 0.07^c^	2.93 ± 0.07^c^

*Note:* Different superscript letters indicate significant differences between groups (*P* < 0.05).

From a physicochemical perspective, aerogenic wasabi sauce samples exhibited significantly higher total acid content and pH values, but lower levels of salt, amino acid nitrogen, moisture, protein, and soluble sugars compared to the normal group. This indicates that there may be microbial contamination in aerogenic wasabi sauce. These microorganisms can break down the sugars and proteins in wasabi mustard while producing carbon dioxide and acidic substances, resulting in increased total acid content and decreased soluble sugar and protein content. Qu et al. (2022) reported that the total acid content in gas‐producing sufu was 9.02% higher than that in normal sufu, while the content of amino acid nitrogen was significantly decreased. These results indicated that package swelling would alter and impair food quality. Nitrogen is an important nutrient element for microbial growth, and the reduction of amino acid nitrogen content may affect the metabolic activities of microorganisms, thereby reducing the production of organic acids and increasing the pH value (Norimatsu et al. 2015). Inorganic salts are an important part of microbial cell structure, which can help maintain the osmotic balance inside and outside the cell during microbial growth, stabilizing the microbial cell structure. Meanwhile, this process may also cause water loss. Wasabi sauce with a bulged package contains a large number of gas‐producing bacteria, which may be the reason for the significant decrease in salt concentration in aerogenic wasabi sauce (Ryu et al. 2021).

### 3.2 Antioxidant Ingredients and Activity Comparison

#### 3.2.1 Comparison of Antioxidant Components

**FIGURE 1:**
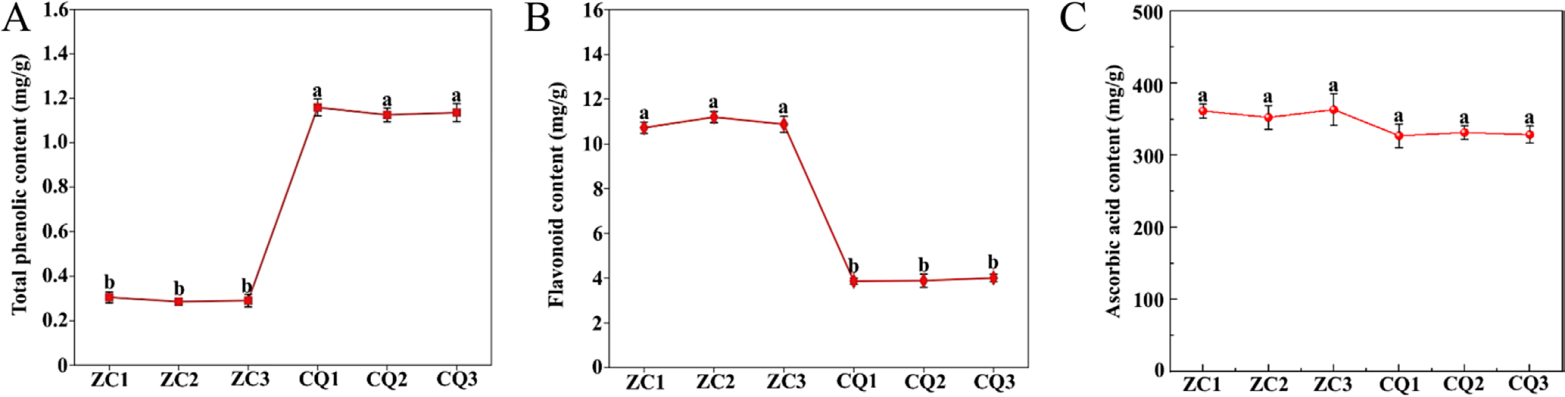
Comparison of antioxidant components between normal and gas‐producing wasabi. (A) Total phenol content; (B) Flavonoid content; (C) Ascorbic acid content. Different superscript letters indicate significant differences among samples (P < 0.05).

Total phenols, flavonoids, and ascorbic acid are common dietary antioxidants, and they have a variety of biological activities, including antioxidative, anti‐inflammatory, and anticancer effects. As shown in Figure 1A, the total phenolic content in aerogenic wasabi sauce is significantly higher than that of normal wasabi sauce, which may be due to the increase in total phenolic content caused by the metabolic activities of some microorganisms in aerogenic wasabi sauce. Previous studies have also confirmed this conclusion, and some microorganisms can release the bound phenolic compounds from the food matrix, converting them into free forms, thereby increasing the total phenolic content (Lin et al. 2024). Compared with normal wasabi sauce, the flavonoid content in wasabi sauce with a bulged package was significantly reduced, and the ascorbic acid content was also reduced (Figure 1B,C). Flavonoids, which contain phenolic hydroxyl groups, and ascorbic acid (vitamin C), a potent reducing agent, are both highly susceptible to oxidative degradation. During spoilage, ascorbic acid is readily oxidized to dehydroascorbic acid and subsequently to non‐antioxidant degradation products (Gupta 2022). Similarly, flavonoids may be broken down by spoilage bacteria to provide carbon sources and energy, leading to a significant decline in their concentration.

#### 3.2.2 Comparison of Antioxidant Activity

**FIGURE 2:**
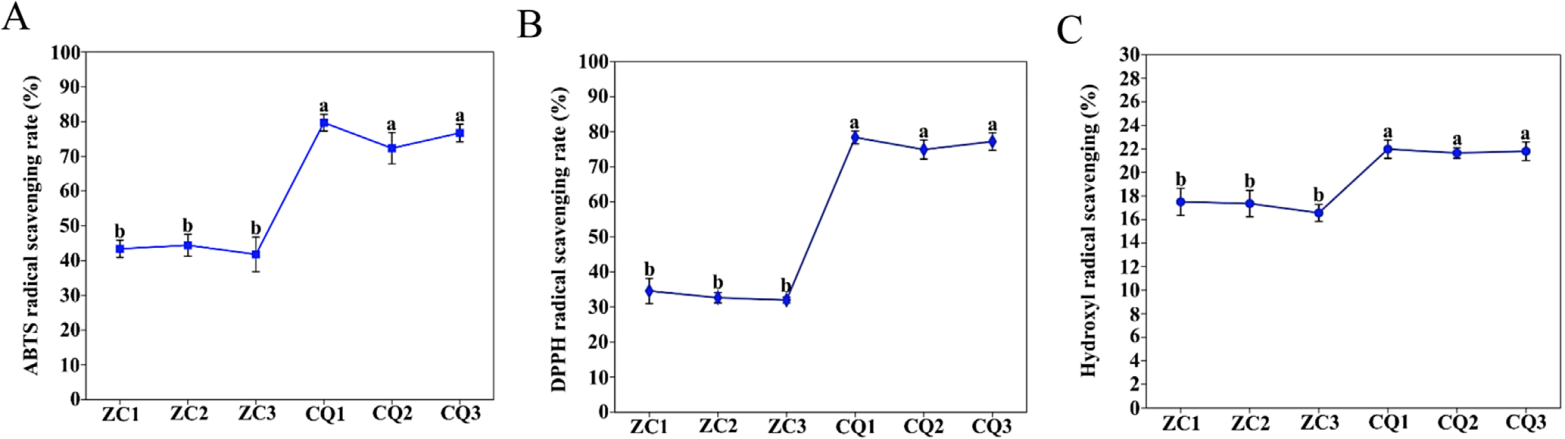
Comparison of antioxidant activity between normal and gas‐producing wasabi. (A) ABTS radical scavenging rate; (B) DPPH radical scavenging rate; (C) Hydroxyl radical scavenging. Different superscript letters indicate significant differences among samples (P < 0.05).

DPPH, ABTS, and hydroxyl radical scavenging capacities were selected as indicators to evaluate the antioxidant activity of wasabi sauce. Typically, antioxidant activity in spoiled food products diminishes significantly and may even be completely lost. However, an unexpected finding in this study revealed that the DPPH, ABTS, and hydroxyl radical scavenging activities of aerogenic wasabi sauce were all significantly enhanced, indicating a much higher antioxidant capacity than in the normal samples (Figure 2). This phenomenon may be attributed to the generation of microbial metabolites during the spoilage process. Certain microorganisms can produce phenolic compounds, flavonoids, or other antioxidant‐active metabolites while degrading food constituents. Although the contents of flavonoids and ascorbic acid were reduced in aerogenic wasabi sauce samples, these compounds may have been transformed into metabolites with greater antioxidant potential (Wang et al. 2021). These newly formed metabolites could act synergistically with existing antioxidants, thereby enhancing overall antioxidant activity. Previous studies have demonstrated that polyphenolic compounds generated by microbial metabolism can synergize with vitamin C to amplify antioxidant capacity in foods (Nowak et al. 2018). Moreover, microbial spoilage may disrupt the structural integrity of the wasabi sauce matrix, causing intracellular antioxidant compounds—previously encapsulated within cells—to be released into the surrounding medium, thereby increasing measurable antioxidant activity.

### 3.3 PCA

**FIGURE 3:**
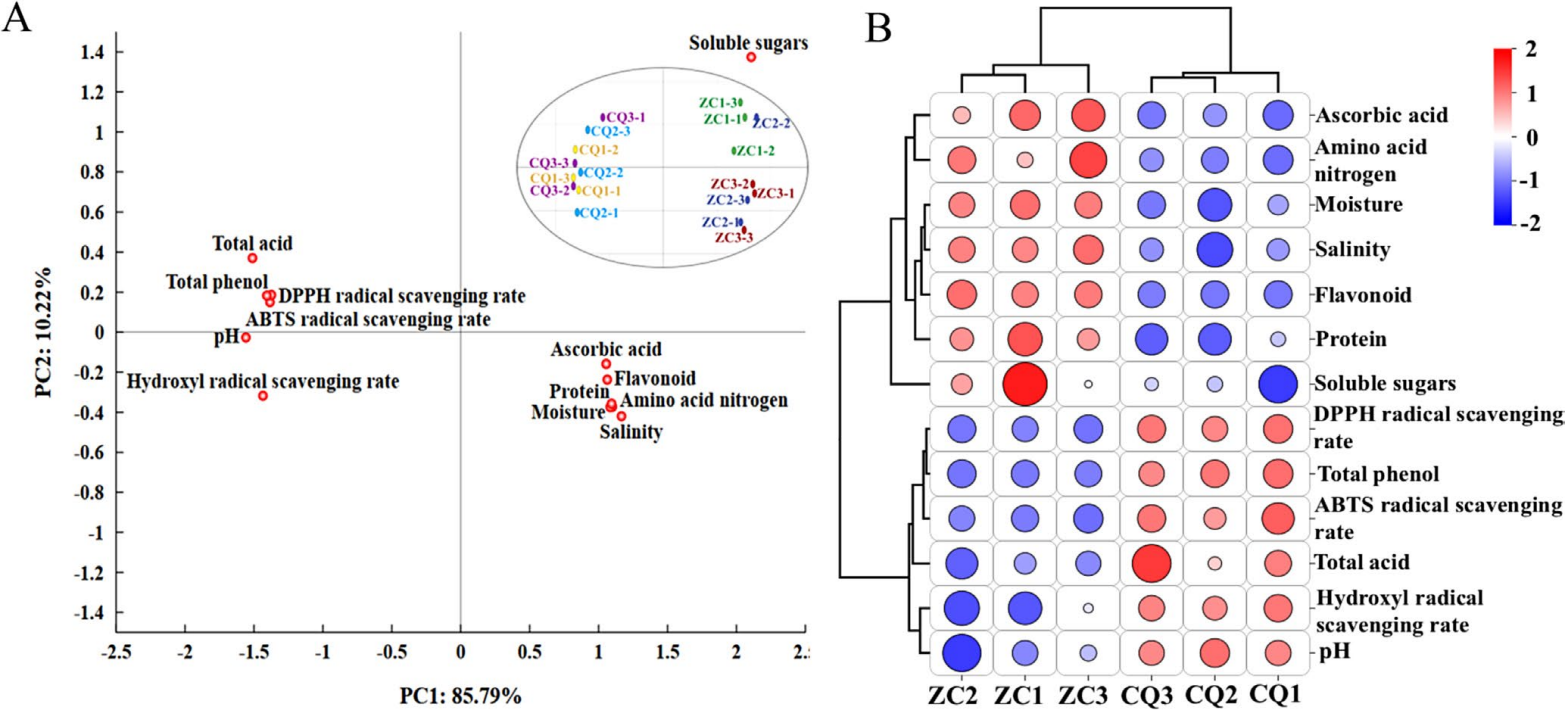
Component plot and hierarchical clustering heat maps of various physicochemical ingredients, functional ingredients, and antioxidant active ingredients. Principal component analysis diagram (A), hierarchical clustering heatmap (B): The red and blue represent positive and negative correlations, respectively; the darker and larger the red circles, the higher the corresponding component content, and the color intensity normalization scale ranges from 2.0 (red circle) to −2.0 (blue circle), indicating the content of each component in the wasabi from the highest to the lowest.

PCA was performed based on physicochemical indices, bioactive compounds, and antioxidant activity. As shown in Figure 3A, the first principal component (PC1) and second principal component (PC2) accounted for 85.79% and 10.22% of the total variance, respectively, explaining a cumulative 96.01% of the data variation. Most quality‐related indicators exhibited high loadings on PC1, suggesting that PC1 plays a dominant role in capturing the variation among samples. The combined score and loading plots indicated that wasabi sauce with bulged packaging was strongly associated with higher total acid content, pH, total phenols, and antioxidant activity. The close clustering of these variables suggests a strong positive correlation among them. In contrast, soluble sugar was positioned distantly from antioxidant activity, implying a negative correlation. This may be due to the conversion of soluble sugars into phenolic compounds during microbial fermentation, or their utilization as metabolic intermediates in amino acid biosynthesis pathways (Han et al. 2017). In addition, the clustering heat map showed that there were significant differences in the physicochemical components, functionally active substances, and antioxidant activity between normal wasabi sauce and wasabi sauce with bulged package. The antioxidant activity of aerogenic wasabi sauce was significantly higher than that of normal wasabi sauce, while the protein content, moisture content, and ascorbic acid content were significantly lower than those of normal wasabi sauce (Figure 3B). This indicated that the antioxidant capacity of wasabi sauce was significantly increased after gas‐producing spoilage, but its quality was significantly reduced. Therefore, despite higher antioxidant activity, consumption of wasabi sauce affected by gas spoilage is not recommended—even if still within its labeled shelf life.

### 3.4 Isolation and Identification of Gas‐Producing Spoilage Bacteria

Four different types of culture media were selected to screen and isolate microorganisms in wasabi sauce with bulged package, with normal wasabi sauce serving as a control. No colony growth was observed on the plates cultured under aerobic conditions. Under anaerobic conditions, no colony growth was also observed on RBM culture media for either group, indicating that mold was not responsible for the package swelling in wasabi sauce samples. The wasabi sauce with bulged package formed six colonies on YPDA media and four colonies on PCA and TSA media, respectively (Figure S1). The low number of colonies was mainly due to the inherent antimicrobial properties of wasabi. Based on differences in colony morphology, isolates were purified, and a total of five distinct strains were obtained, designated QJL1 to QJL5 (Figure S2).

**FIGURE 4:**
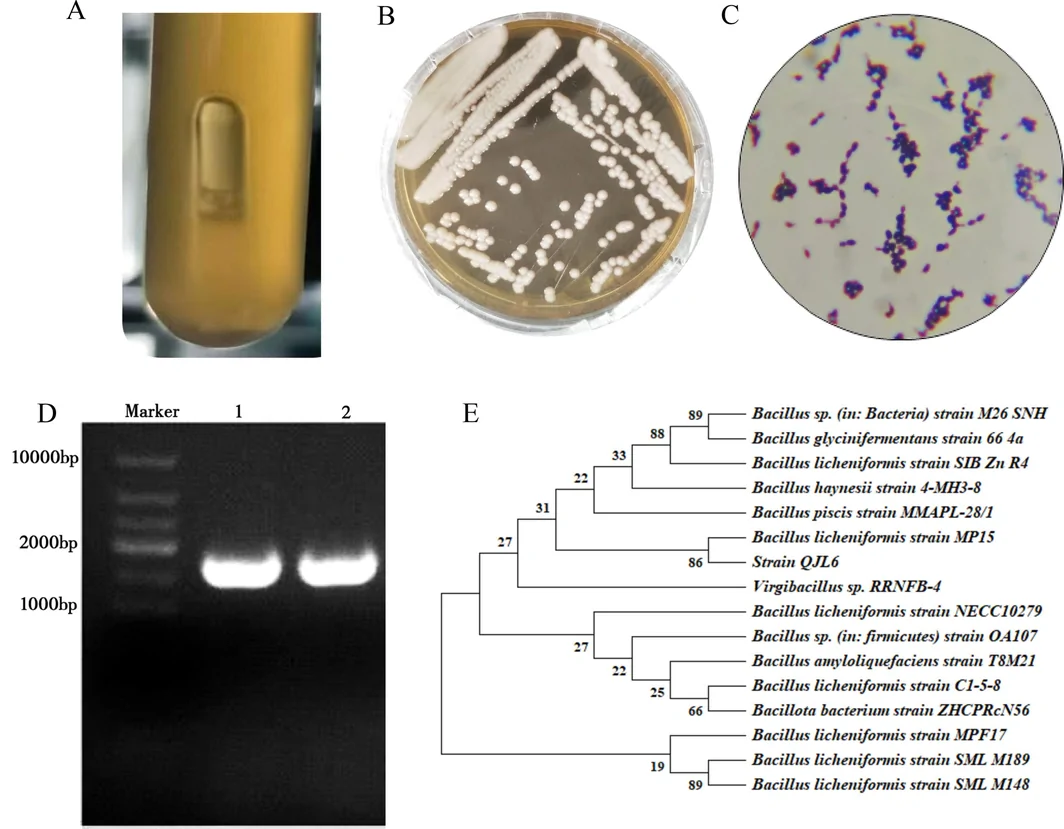
The Durham tube test (A), colony morphology (B), Gram stain microscopy images (C), electrophoresis image of 16S rDNA PCR amplification (D), phylogenetic tree of strain QJL3. The medium used in the gas‐producing ability test was YPDA liquid medium, and the plates used for morphological observation were YPDA agar plates, which were both cultivated in anaerobic conditions.

To determine which strain was responsible for gas production, each isolate was inoculated into the appropriate liquid medium containing a Durham tube. Within 24 h, only strain QJL3 produced visible gas accumulation in the Durham tube (Figure 4A and Figure S3), prompting further investigation of this strain. After 48 h of incubation at 37°C on YPDA agar, colonies of QJL3 appeared round, milky white, 2–4 mm in diameter, with a matte surface and the development of wrinkles upon extended incubation (Figure 4B). Scanning electron microscopy revealed that the bacterial cells were rod‐shaped and Gram‐positive (Figure 4C). According to results from the automated microbial identification system (VITEK 2), strain QJL3 was identified as *B. licheniformis* . The strain exhibited the ability to metabolize glucose, galactose, maltotriose, and mannose to produce acid and gas. It also produced enzymes such as l‐asparaginase, leucine arylamidase, and phenylalanine arylamidase. Additionally, it demonstrated resistance to kanamycin, oleandomycin, and polymyxin B (Table 3). While these biochemical and physiological characteristics provided strong preliminary evidence, they were not sufficient for definitive identification.

**TABLE 3:** Physiological and biochemical analysis of strain QJL3.

Sugar utilizing ability	Results	Enzyme‐producing properties	Results	Others	Results
D‐glucose	+	β‐Xylosidase	−	Putrescine assimilation	+
Maltotriose	+	Glycine arylamine	−	Methyl‐α‐d‐glucopyranosylation	+
d‐galactose	+	l‐lysine arylamine	−	d‐mannitol	+
d‐Mannose	+	l‐aspartate arylamine	+	Resistant to kanamycin	+
l‐rhamnose	+	Leucine arylamine	+	Resistant to oleandomycin	+
d‐ribose	+	Phenylalanine arylamidase	+	Esculin hydrolysis	+
d‐trehalose	+	α‐mannosidase	−	Resistant to polymyxin B	+
d‐melezitose	+	β‐Mannose enzyme	−	Grown in 6.5% NaCl	+

Therefore, molecular identification was carried out via 16S rDNA sequencing. The 16S rDNA fragment of strain QJL3 was successfully amplified (Figure 4D) and sequenced. The sequence was submitted to NCBI BLAST for homology comparison, and a phylogenetic tree was constructed using the neighbor‐joining method (Figure 4E). The results revealed that the 16S rDNA sequence of QJL3 shared 96% similarity with *B. licheniformis* , clustering in the same clade. Based on morphological, biochemical, and molecular data, strain QJL3—isolated from the aerogenic wasabi sauce—was conclusively identified as *B. licheniformis* .

### 3.5 Environmental Tolerance of Gas‐Producing Bacteria

**FIGURE 5:**
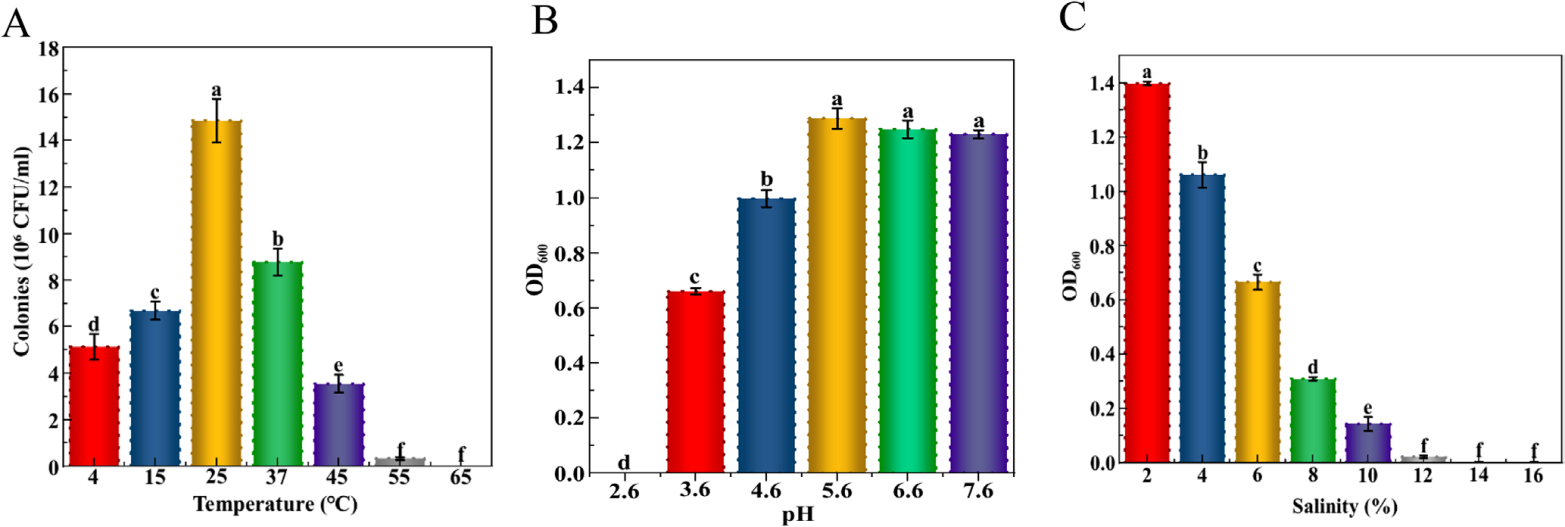
Resistance of *Bacillus licheniformis* QJL3 to different environmental stresses: (A) Temperature, (B) pH, and (C) salinity.

The environmental adaptability of *B. licheniformis* QJL3 was evaluated under various stress conditions, including moderate heat, low pH, and high salinity. The strain exhibited optimal growth at 25°C, and it can survive after being treated at 4°C and 55°C for 10 min (Figure 5A). Growth was markedly inhibited under strongly acidic conditions (Figure 5B); bacterial concentration remained low at pH 3.6, and increased progressively with rising pH. However, when the pH value was raised to a neutral environment, the growth of the strain tended to be stable, and pH 5.6 was identified as the optimal pH for this strain. Salt not only contributes to the flavor profile of wasabi sauce but also plays a crucial role in suppressing microbial contamination (Tang et al. 2024). As the salinity increased, the growth of *B. licheniformis* QJL3 was gradually inhibited (Figure 5C). However, *B. licheniformis* QJL3 can still grow and produce gas in YPDA liquid medium with a salinity of 12%, and its growth is completely inhibited at a salinity of 14%. Collectively, these findings suggest that *B. licheniformis* QJL3 is tolerant to high salt concentrations and moderate heat but sensitive to highly acidic conditions. Niu et al. (2018) found that *B. licheniformis* FHYM 8 is the primary gas‐producing spoilage bacterium in fava bean paste, and this facultative anaerobe can tolerate a high osmotic environment (15% salinity) and acidic conditions (pH = 3.8). The spores of *Bacillus* have a dormant trait. Under appropriate temperatures or specific stimuli, the dormant spores begin to germinate, consuming sugars in the system in a relatively anaerobic environment, producing acids and alcohol, and generating CO_2_, which leads to swelling of containers or packages. The above results indicated that *B. licheniformis* QJL3 was responsible for the swelling of wasabi sauce packaging.

### 3.6 Inhibitory Effects of Nisin, Preservatives, and Salt on *B. licheniformis* QJL3

**FIGURE 6:**
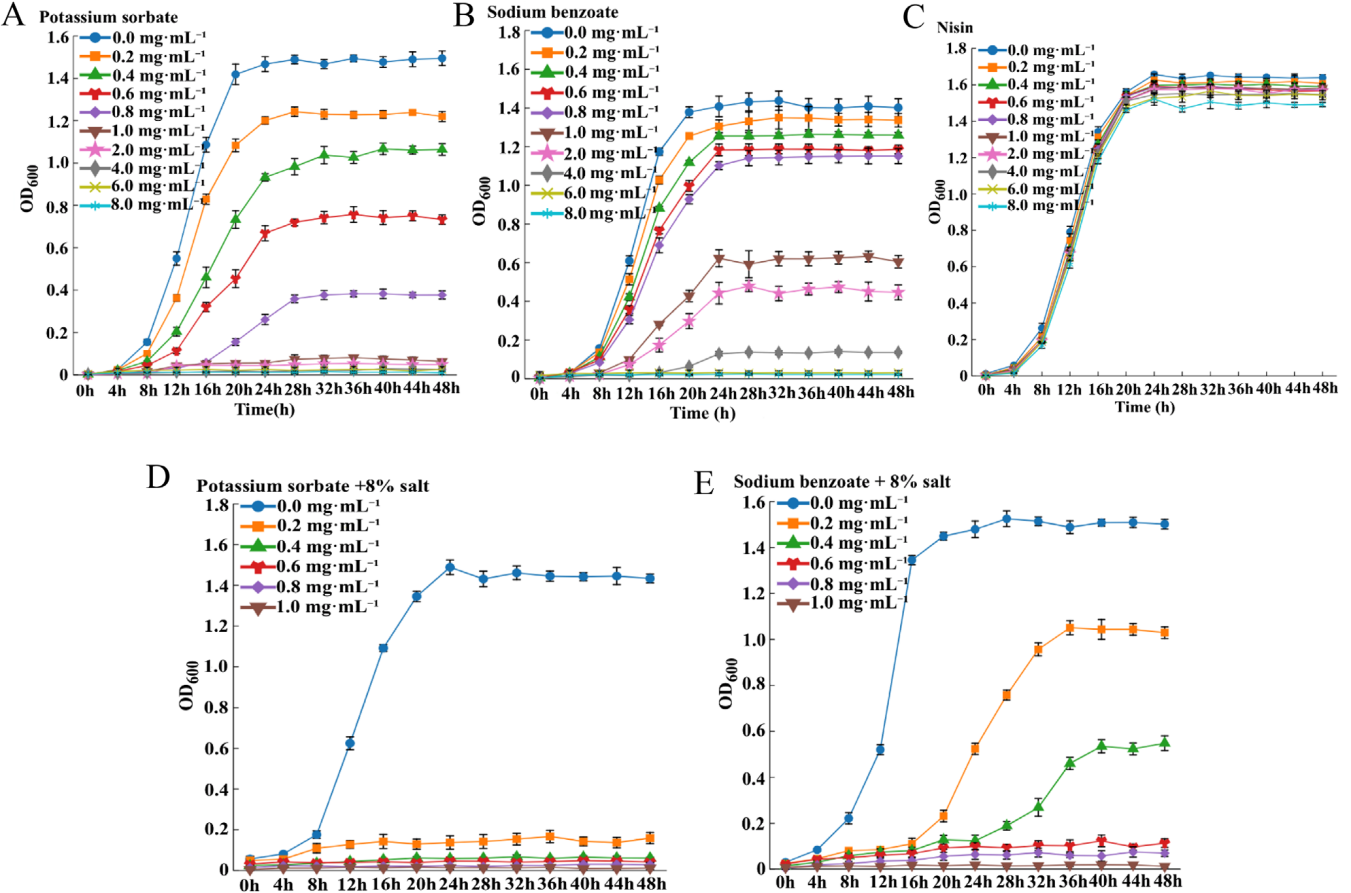
Growth curves of *Bacillus licheniformis* QJL3 under different concentrations of potassium sorbate (A), sodium benzoate (B), nisin (C), and growth curves of *B. licheniformis* QJL3 under different concentrations of potassium sorbate (D) and sodium benzoate (E) when 8% salt was used.

Due to the semi‐solid nature of wasabi sauce, direct assessment of *B. licheniformis* QJL3 growth within the product matrix is challenging. Therefore, the antibacterial effects of nisin, preservatives, and salt were tested in YPDA liquid medium, and the MIC was determined. Potassium sorbate and sodium benzoate showed varying degrees of inhibition effects on the growth of *B. licheniformis* QJL3 under salt‐free conditions, with MIC being 1 and 4 mg/mL, respectively (Figure 6A,B). However, nisin had no inhibitory effect on *B. licheniformis* QJL3 (Figure 6C). When the salt concentration was increased to 8%, the MICs of potassium sorbate and sodium benzoate were reduced to 0.2 and 0.6 mg/mL, respectively (Figure 6D,E). Moreover, the MIC of potassium sorbate at an 8% salt concentration (0.02 mg/mL) was lower than the minimum standards in China, the European Union, and the United States. Potassium sorbate has been widely used in milk, fruit juice, ketchup, soy sauce, and meat products by changing the morphology of cell membranes and inhibiting their transport functions, thereby affecting the metabolic activity and growth of microorganisms (Awaad et al. 2023). After entering the human body, potassium sorbate is metabolized and excreted in the form of carbon dioxide and water, posing virtually no harm to humans and having no adverse effects on food quality. The Food and Agriculture Organization of the United Nations has recommended potassium sorbate as a safe and efficient food preservative (Tango et al. 2016). Liang et al. found that sorbate could effectively prevent the growth of *Clostridium perfringens* spores and inhibit spore germination (Liang et al. 2023). Aouadhi et al. found that after 0.2% potassium sorbate treatment for 6 h, *Bacillus thermophilus* in milk decreased by 1.1 log, which could effectively inhibit the germination and growth of spores (Aouadhi et al. 2015). Therefore, the combination of potassium sorbate (0.2 mg/mL) and 8% salt can effectively inhibit the growth of strains in YPDA liquid medium. However, given the complexity of real food systems, the antibacterial effect of potassium sorbate may gradually weaken, and higher doses may be required to ensure antibacterial efficacy. Nevertheless, the results of this study provide an effective strategy for wasabi sauce producers to inhibit gas‐producing spoilage bacteria, which can help improve the microbiological stability and shelf life of the product.

## 4 Conclusion

In this study, the sensory, physicochemical indexes, and antioxidant activity of wasabi sauce with bulged package and normal wasabi sauce were analyzed. The aerogenesis defect samples exhibited packaging expansion, darker coloration, reduced levels of protein, amino acid nitrogen, and soluble sugars, along with elevated total acid content and antioxidant activity. In addition, *B. licheniformis* QJL3 was confirmed to be the main microorganism causing the aerogenesis defect of wasabi sauce. It demonstrated notable tolerance to moderate heat, low pH, and high salinity, confirming its ability to survive under wasabi sauce storage conditions and induce packaging swelling. In YPDA medium, the combination of potassium sorbate and edible salt significantly inhibited its growth. While salinity above 14% could suppress microbial proliferation, ongoing public health initiatives advocating for reduced sodium intake necessitate alternative strategies. This study demonstrates that an 8% salt concentration—significantly lower than the conventional 10%–12%—in combination with potassium sorbate effectively meets microbial control requirements while aligning with public health sodium reduction policies. The research results provide a theoretical foundation and practical strategy for wasabi sauce manufacturers to mitigate aerogenesis defects, enhance product quality, and minimize economic losses. Future research should focus on determining the optimal dosage of potassium sorbate under real production conditions to ensure both product stability and the preservation of traditional sensory characteristics.

## Author Contributions

**Xiao Liu:** conceptualization, writing – original draft, writing – review and editing, methodology, investigation, software, data curation, formal analysis, validation, visualization. **Yun‐Guo Liu:** funding acquisition, project administration, resources, supervision. **Qing‐Dian Han:** investigation, formal analysis, supervision, resources. **Qin‐Guo Xu:** formal analysis, data curation. **Hong Li:** investigation, software, methodology. **Weiwei Wu:** methodology, supervision, conceptualization.

## Funding

This work was supported by the State Administration for Market Regulation Science and Technology Plan Project (2024MK221) and the Innovation team of the introduction and education plan for young and innovative talents in Shandong Provincial University (2021QCYY007).

## Conflicts of Interest

The authors declare no conflicts of interest.

## Supporting information


**Figure S1:** Isolation of microorganisms in wasabi sauce.
**Figure S2:** Purification of isolated strains.
**Figure S3:** Gas production verification test results.

## Data Availability

The data that support the findings of this study are available from the corresponding author upon reasonable request.
