# Supplementary material for: Isolation and Identification of Gas‐Producing Spoilage Bacteria in Wasabi Sauce and Effective Methods for Controlling Their Growth
Source: Food Sci Nutr. 2026 Sep 23;14(9):e71750. doi: 10.1002/fsn3.71750 (PMC13599355; doi:10.1002/fsn3.71750)
Supplement: Supplementary file 1 [file FSN3-14-e71750-s001.docx]

**Supplementary data**

A

B

Figure 1 Isolation of microorganisms in wasabi sauce

Figure 2 Purification of isolated strains

Figure 3 Gas production verification test results
